# Bile acid binding protein: a versatile host of small hydrophobic ligands for applications in the fields of MRI contrast agents and bio-nanomaterials

**DOI:** 10.5936/csbj.201303021

**Published:** 2013-12-08

**Authors:** Katiuscia Pagano, Simona Tomaselli, Serena Zanzoni, Michael Assfalg, Henriette Molinari, Laura Ragona

**Affiliations:** a Istituto per lo Studio delle Macromolecole, CNR, via Bassini 15, 20133 Milano, Italy; b Dipartimento di Biotecnologie, Università degli Studi di Verona, Strada Le Grazie 15, 37134, Verona, Italy

## Abstract

During the last decade a growing amount of evidence has been obtained, supporting the role of the beta-clamshell family of intracellular lipid binding proteins (iLBPs) not only in the translocation of lipophilic molecules but also in lipid mediated signalling and metabolism. Given the central role of lipids in physiological processes, it is essential to have detailed knowledge on their interactions with cognate binding proteins. Structural and dynamical aspects of the binding mechanisms have been widely investigated by means of NMR spectroscopy, docking and molecular dynamics simulation approaches. iLBPs share a stable beta-barrel fold, delimiting an internal cavity capable of promiscuous ligand binding and display significant flexibility at the putative ligand portal. These features make this class of proteins good scaffolds to build host-guest systems for applications in nanomedicine and nanomaterials.

## Introduction

The intracellular lipid binding protein (iLBP) family [1, 2], composed of phylogenetically related low molecular weight proteins, includes the subclass of cytosolic bile acid binding proteins (BABPs), the focus of this review. BABPs, similarly to the other members of the iLBP family, are characterized by the presence of approximately 125 amino acid residues folded into ten antiparallel beta-strands that form a clamshell-like structure, capped by a pair of alpha-helices, delimiting an internal cavity for ligand binding [3]. It is widely accepted that the main function of BABPs is to bind and shuttle bile acids (BAs) across the cytosol, facilitating their intracellular solubilisation and trafficking between membranes [4, 5]. Bile acids not only play an important role in lipid absorption but also have the ability to regulate gene expression by serving as ligands for several nuclear receptors, such as farnesoid-X-receptors (FXR), liver-X-receptor (LXR) and G protein-coupled receptors (GPCR) [6–8]. FXR and LXR serve as BAs targets for the regulation of BABPs expression level in the cells [9, 10]. The interaction of GPCR with BAs activates a signalling cascade for the regulation of hepatic lipids, glucose, and energy homeostasis [8]. It should also be mentioned that in addition to the beta-clamshell protein family emphasized in this review and the bile acid binding receptors, another group of proteins is implicated in binding and hepatic transport of bile acids: the hydroxysteroid dehydrogenases, members of the aldo-keto reductase superfamily [11].

The precise role of cytosolic bile acid binding proteins is not completely clarified, however few *in vivo* experiments have been performed to assess the role of ileal BABP using mice with global FXR deficiency, which are also deficient in ileal BABP [6, 12]. The finding was that this protein is not required for the liver-small intestine circulation but its exact role in bile acid transport and metabolism could not be properly assessed because FXR is needed for appropriate expression of many genes involved in bile acid transport and metabolism. The creation of a mouse line that specifically lacks ileal BABP was recently reported [13], demonstrating that ileal BABP is involved in the apical to basolateral transport of bile acids in ileal enterocytes, and is vital for the maintenance of bile acid homeostasis in the liver-small intestine circulation in mice.

Hereditary and acquired defects of BA transporters are involved in the pathogenesis of several hepatobiliary disorders including cholestasis, gallstones, fatty liver disease and liver cancer, but also play a role in intestinal and metabolic disorders beyond the liver [14]. In accordance, the study of the mechanisms of bile acids binding and release from their protein carriers is essential to understand intracellular lipid transport, and represents an important step towards the identification of new therapeutic targets for the prevention and treatment of metabolic diseases. The broad-spectrum binding ability for hydrophobic or amphiphilic small molecules displayed by liver BABPs makes these proteins suitable candidates to escort a variety of physiological and exogenous ligands across the hepatocytes [15]. BABPs can be viewed as natural nano-carriers, controlling intracellular drug distribution. Thus this versatile and abundant polypeptide scaffold has become an attractive target for binding molecular probes for applications in biomedicine and optoelectronics.

In the following paragraphs the binding of endogenous and exogenous ligands will be discussed in detail.

## 1. BABP as host of endogenous ligands: bile acid pool

**Figure 1:**
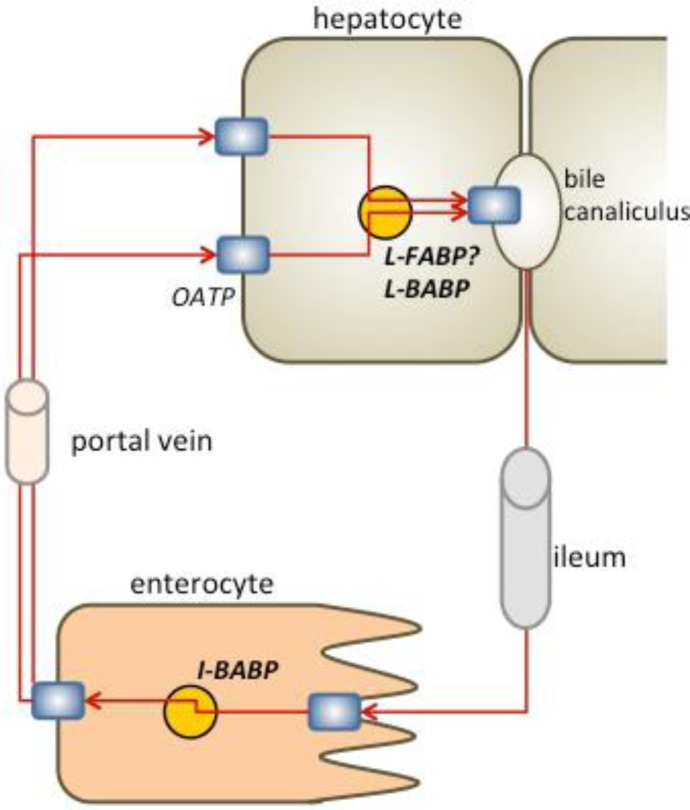
Schematic representation of the liver-small intestine circulation of bile acids. The cytosolic carriers ileal and liver bile acid binding proteins (I-BABP and L-BABP) are shown as yellow circles. In mammalian hepatocytes the liver fatty acid binding protein (L-FABP) may act as bile acid carrier [18, 26]. Receptor systems, mediating bile salts import and excretion from the cell, are shown as blue diamonds. Organic anion transport polypeptide (OATP) is labelled. Red arrows indicate the direction of bile salts circulation.

The liver-small intestine circulation, namely the body recycling system for bile acids, requires: (i) a receptor system, that uptakes bile salts and translocates them into the cell; (ii) a cellular bile salt binding protein, that moves them across the cell; and (iii) an exit system, which regulates bile salts excretion [5, 16] (Figure 1). By studying the human and chicken proteins, we showed that cytosolic bile salt binding proteins exist and operate in parallel in the liver and ileum of both mammalian and non-mammalian species [16]. We indeed identified a non-mammalian ileal BABP that acts as bile acid transporter in the ileum, and, together with the liver BABP, ensures the circulation of bile acids in liver and intestine. We also proved that the human liver fatty acid binding protein (FABP) may act as a bile acids carrier, contributing, together with the known human ileal BABP [17], to the regulation of bile acid metabolism [16, 18].

BABPs and liver FABPs display an unusual ligand binding stoichiometry, within the iLBP family, being able to host two ligands in the large internal binding cavity (roughly 20 x 18 x 8 Å wide). NMR structures of BABPs binding either two copies of the same ligand or two different ligands have been reported only for chicken liver and ileal complexes [19–21]. X-ray structures have been reported for the liver proteins from chicken, zebrafish, toad, axolotol and for the ileal zebrafish protein, bound to two copies of the same bile acid ligand [22–25].

Recent studies addressing the binding stoichiometry of rabbit ileal-BABP, using calorimetry combined with MS [27] and of zebrafish ileal-BABP by X-ray crystallography [23], suggested that these proteins have the capability of binding one or two ligands on the molecular surface in addition to the two bile acids bound in the inner cavity. However, the most interesting feature is the highly cooperative binding of some BABPs towards glycocholic (GCA) and glycochenodeoxycholic acids (GCDA) [28, 29], which are the major components of the bile acid pool and also the ligands of the farnesoid X receptor (FXR).

**Figure 2:**
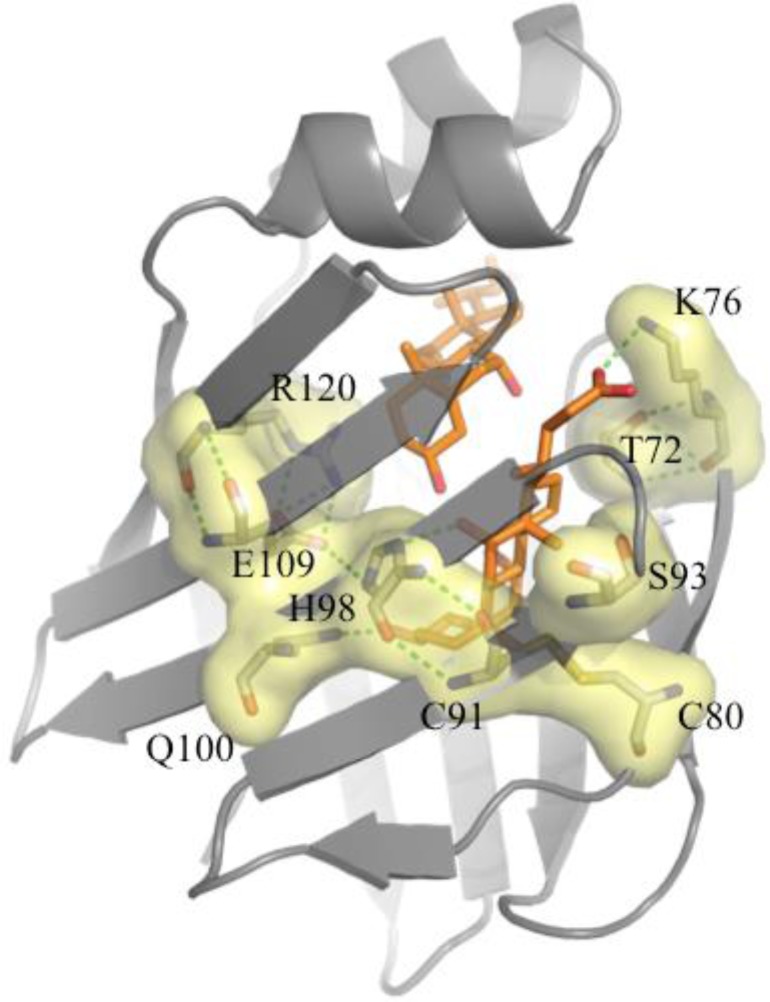
Polar spine. Polar residues (represented as sticks with the corresponding label) connected by H-bonds (green dotted lines) define a polar spine, represented by a pale yellow surface. The two bound bile salts are represented with orange sticks.

^13^C, and/or ^15^N-enriched GCA and GCDA were used in two-dimensional heteronuclear NMR titration studies to monitor the occupancy of each binding site in both ileal and liver BABPs [29, 30]. These investigations highlighted the excellent resolving power of NMR to detect recognition events in a site-specific manner making it possible to assess the occurrence of microscopic cooperativity. Human ileal BABP and chicken liver BABP were shown to interact with GCA and GCDA, respectively, with modest intrinsic affinity and a high degree of positive cooperativity (the microscopic cooperativity factor exceeding 10^3^, possibly the largest value ever measured for a ligand-protein interaction) [28, 29, 31]. The structural basis for the observed cooperativity is proposed to result from allostery, where the binding of the first ligand is energetically communicated to the second site through a conformational change in the protein [32–35]. The latter can be detected by NMR using reverse labelling schemes with respect to those employed to monitor binding sites occupancies. In fact the chemical shift changes of protein ^15^N-amide resonances, upon addition of unlabelled ligand, allow for the detection of protein structural rearrangement in an aminoacid-specific manner. The binding energetics suggests that BABP may function as a ‘regulated sponge’, permissive at low ligand concentrations but protective at higher levels [34]. Much work has been then devoted to unravel the mechanism of allostery, describing the structural determinants of ligand binding and evaluating the role of protein dynamics in ligand recognition [30, 31, 36]. Specifically one highly conserved buried histidine (H98) was indicated to play a central role in establishing a network of hydrogen bonds and salt bridges among buried residues, defining a sort of continuous polar ‘‘spine’’ connecting remote strands of the liver apo protein (Figure 2) [20, 32]. Indeed a mutation of H98 was shown to disrupt the energetic communication functional to efficient binding [29].

The information obtained for the chicken liver protein was essential in the unravelling of the determinants of cooperativity in the chicken ileal protein [37]. Employing a gain-of-function approach, the non-cooperative chicken ileal BABP was turned into a cooperative protein, designing appropriate mutants and showing, on the basis of NMR data coupled to molecular dynamics simulations, that the cooperative binding mechanism requires the presence of few latch residues, located in the inner protein cavity, that click into their final conformation upon ligand addition and stabilise an extended communication network necessary for establishing energetic coupling between binding sites [37].

In this context, 2D line shape analysis [38] applied to a titration experiment of liver BABP, in the presence of different concentrations of the binding partner (GCDA), provided information on the effect of ligand binding on the two adjacent nuclei (^1^H and ^15^N) throughout the entire titration. The line shape simulation of cross-sections corresponding to each single residue allowed the extraction of the kinetic parameters and the investigation of the binding mechanism. A group of residues were identified, mostly distributed in the β-barrel, characterized by a low-intensity signal for the apo form, consistent with the presence of conformational averaging, occurring on the µs-ms timescale. Thus an equilibrium P⇔P*, with P* representing a binding-conformation, was proposed as the first binding step [39]. Relaxation dispersion (RD) experiments further supported the presence of a conformational selection mechanism [39, 40].

**Figure 3:**
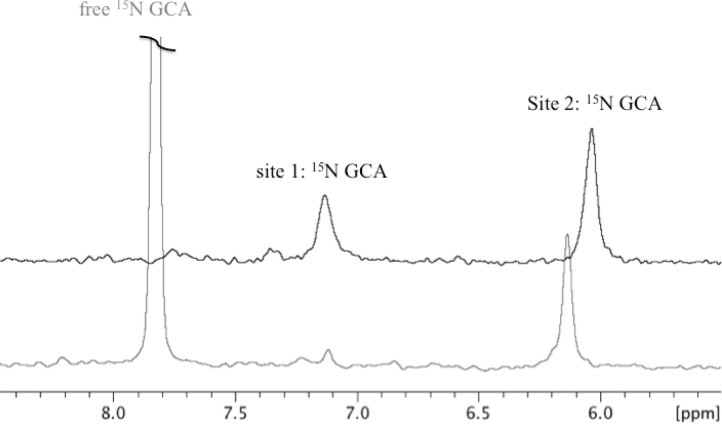
Displacement of labelled ^15^N-GCA in site 1 by unlabelled (NMR invisible) GCDA, showing the higher affinity of this site for GCDA. First increment of the 2D ^1^H-^15^N-HSQC spectra collected on T91C-BABP:^15^N-GCA 1:2 molar ratio (black upper spectrum) and on T91C-BABP:^15^N-GCA:u-GCDA 1:2:2 molar ratio (grey lower spectrum); the resonances corresponding to the free ligand and to ligands bound to site 1 and 2 are indicated. The observed difference in the chemical shifts of the ^15^N amide of GCA bound to site 2, in the two spectra, is due to the different environment provided by GCA or GCDA in site 1. The black spectrum is vertically translated with respect to the grey one for clarity reasons.

Also highly interesting is the capability of these proteins to discriminate between GCA and GCDA, which differ only for a single hydroxyl group at position C-12 of their steroid ring system. It was shown that when the human ileal protein is incubated with a mixture of the two bile salts, GCA binds nearly exclusively to one site, while GCDA binds nearly exclusively to the other site [33]. In the case of the chicken liver scaffold, the presence of a disulphide bridge was required to confer site selectivity to the protein [20]. Indeed sequence analysis of non-mammalian chicken liver BABP (access code P80226 in Swiss-Prot) reported the presence of a cysteine residue at position 80 and of a threonine residue at position 91. An annotated conflict is reported for position 91, corrected to a cysteine, capable of forming an intramolecular disulphide bridge with cysteine 80. Alignment of non-mammalian liver BABPs shows that a cysteine in position 91 is present in nearly all aligned sequences, with the exception of lizard and axolotl [22]. Both chicken liver proteins, with and without the disulphide bridge, were produced and their dynamics and interactions with endogenous ligands studied [20, 41]. Only the protein endowed with the disulphide bridge displayed site-selectivity, as clearly shown by the first trace of ^1^H-^15^N HSQC experiments (Figure 3), whereby a sample of T91C-BABP:^15^N-GCA complex (1:2) (Figure 3, black line) after addition of unlabelled GCDA (u-GCDA) (Figure 3, grey line) showed the disappearance of the resonance corresponding to site 1, occupied by u-GCDA. The intensity of the site 2 resonance was substantially unaffected, indicating that ^15^N-GCA remains in site 2. The intense resonance occurring at 7.9 ppm, corresponds to the free ^15^N-GCA, expelled from site 1.

**Figure 4:**
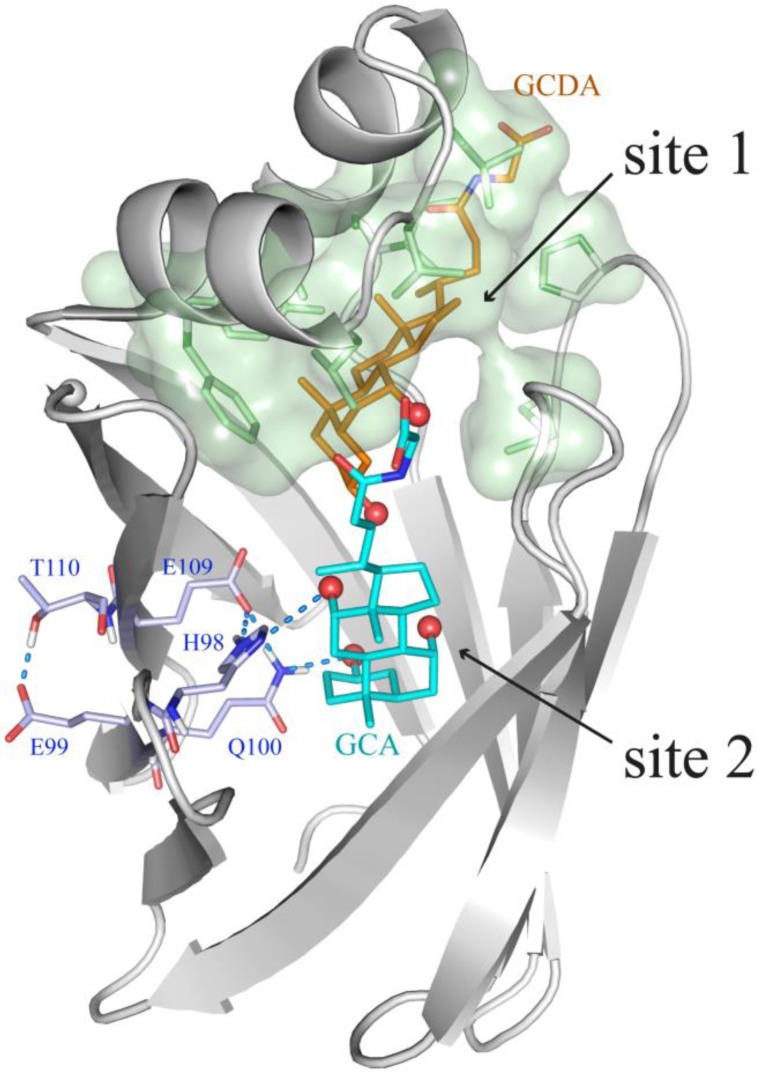
NMR structure of the ternary complex of BABP binding two different ligands, namely GCA (cyan) at site 2 and GCDA (orange) at site 1 (PDB id: 2LFO). The OH groups of the two bile salts are highlighted as red spheres. H98, E99, Q100, E109 and T110 side chains are shown as light blue sticks and their H-bonds are represented as dashed blue marine lines. BABP residues (Y14, F17, L18, L21, L23, L27, A31, I34, P54, M73 and I111) side-chains, defining the highly hydrophobic surface, which host GCDA, are depicted in green.

The NMR structure of the ternary complex with two different ligands, T91C-BABP:GCA:GCDA, the first reported for this protein family (PDB id: 2LFO), showed that the protein with the disulphide bridge, preferentially hosts GCA in site 2 (Figure 4), thanks to the stabilizing interactions established between the side chain of the highly conserved H98 and the C-12 hydroxyl group, absent in GCDA. The more hydrophobic GCDA is bound to site 1, which displays a more hydrophobic character [20]. The presence of a S-S link between adjacent strands was shown to favour specific rotameric states for residues E99, Q100 and E109, allowing for the onset of an extended intramolecular hydrogen bond network and the consequent stabilization of the side-chain orientation of the buried H98, thus capable of anchoring GCA.

## 2. BABP as host of a bile acid based Gd chelate for the development of hepatospecific MRI contrast agent

Interest in interactions involving iLBPs has grown during the past few years essentially because these proteins are considered efficient carriers of lipophilic drugs in the cytoplasm and they have been proposed as pharmaceutical targets [15]. The acquired knowledge on BABP/lipid interactions has served for the development of hepatospecific contrast agents (CAs) for magnetic resonance imaging (MRI). A high intrinsic contrast is often obtained using CAs based on gadolinium(III) complexes, molecules endowed with favourable electronic and magnetic properties [42]. Despite the elevated number of clinical MRI scans using CAs, sensitivity, specificity, and latent toxicity still remain challenging problems [43]. The development of new MRI contrast agents endowed with tissue selectivity constitutes an important step forward in the field. In the case of liver-targeted CAs, the active lipid internalization into hepatocytes, mediated by organic anion transport polypeptides (OATP) [44], provides a convenient route that can be exploited to obtain efficient uptake of CAs, e.g. constituted by lipophilic Gd(III) complexes. This approach avoids the limitations of nonspecific tissue uptake of low molecular weight gadolinium chelates. These systems are highly diagnostic of specific hepatic malignancies since OATPs are not expressed in the basolateral membrane of some hepatoma cell lines^45^. A number of potential CAs were synthesized by conjugating bile acid moieties to a DTPA gadolinium chelating unit, separated by different spacers [46]. Noticeably, cellular uptake measurements in hepatocytes and tumoral cells showed a clear differential uptake of two related Gd-DTPA-based conjugates of cholanoic and deoxycholic acids [47]. Different modifications of these compounds were tested by relaxometry, i.e. the analysis of proton relaxation enhancement due to the presence of the paramagnetic species, and other NMR experiments for their binding to liver BABP *in vitro* (vide infra) [46]. Indeed it is expected that bile acid-based molecules, such as the described CAs, should interact with these highly abundant transporters inside the cell.

**Figure 5:**
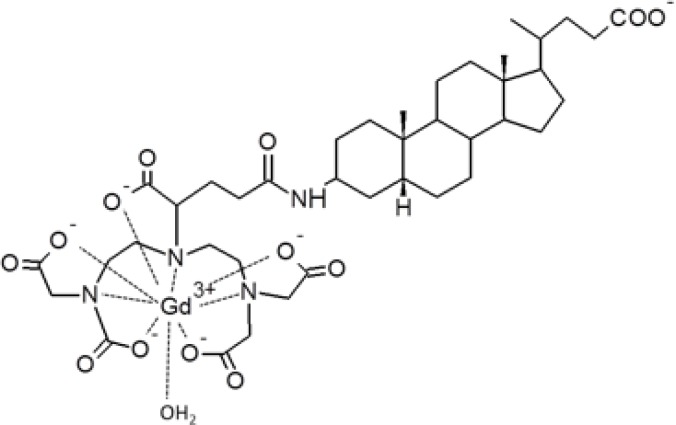
Chemical structure of the amphiphilic bile acid-based Gd(III) complex (Gd-BA).

Liver BABP was shown to be able to bind a single molecule of bile-acid modified Gd complex (Gd-BA) (Figure 5) with dissociation constants in the sub-micromolar range, as determined by both relaxometric and NMR titration experiments [47]. Importantly, the relaxivity values (defined as the relaxation enhancement of water protons in the presence of the paramagnetic complex at 1 mM concentration) of Gd-BAs increased up to three-fold compared to the value measured for the isolated compound, due to favourable relaxation properties in the protein-ligand adducts. In order to provide the structural basis for an improved design, the mode of binding of a Gd-BA to liver BABP was investigated. To obtain structural restraints for the positioning of the Gd chelate inside the protein, a diamagnetic dilution with yttrium complexes was necessary to avoid signal cancellation by the strongly paramagnetic Gd [46]. This substitution, due to the very similar properties of the two elements, is not expected to introduce significant perturbations in the molecular structure and solution dynamics of the complex. Thus protein:ligand 1:5 ratios were employed, where the following relative amounts of protein: diamagnetic ligand (Y^3+^): paramagnetic ligand (Gd^3+^) were employed: 1:4.76:0.24 and 1:4.55:0.45.

**Figure 6:**
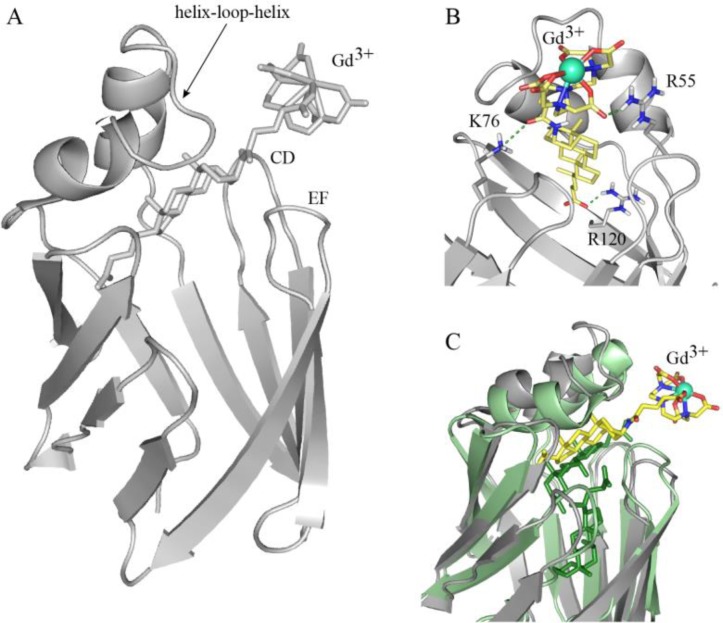
BABP-Gd-BA host-guest complex. A) NMR structure of the lipid functionalised gadolinium chelate in complex with BABP (PDB 2K62). Loops defining the protein entrance to the cavity are labelled. B) Polar residues establishing hydrogen bonds with the ligand. C) Superposition of the BABP-Gd-BA complex (grey) with the protein in the presence of endogenous bile salts (PDB 2JN3) (green), indicating the opening of the helix-loop-helix motif at the protein open end. Bound Gd-complex is represented with yellow sticks, while the two bound GCDA are shown as green sticks.

The analysis of paramagnetic-induced protein NMR signal intensity changes was initially employed as a qualitative tool to identify protein “hot spots” defining the binding site, which is located at the protein portal region. The data were also analysed quantitatively for the precise metal-ion-center positioning in the protein-ligand supramolecular adducts. Thus 19 distances were derived from paramagnetic relaxation enhancements. Other 14 intermolecular NOE-derived distances were obtained from 3D edited/filtered NOESY experiments. All these unambiguous restraints were used together with ambiguous interaction restraints (AIR), derived from chemical shift perturbations upon ligand binding, to locate the Gd-BA in the protein binding sites by using a data-driven docking method (HADDOCK) and derive the final structure (PDB id: 2K62) (Figure 6).

It was found that Gd-BA binds to the “upper” part of the large hydrophobic protein cavity (Figure 6A). The steroid moiety becomes substantially buried inside the cavity, surrounded by protein hydrophobic residues, while the bulky hydrophilic ion chelating moiety protrudes at the exterior of the protein. A few positively charged residues (R55, K76 and R120) establish polar contacts with the ligand, stabilizing the complex (Figure 6B). A structural comparison between the BABP-Gd-BA adduct and BABP in complex with endogenous ligands highlights conformational changes in correspondence of the so-called protein open end region (Figure 6C), which controls the access to the binding cavity. Specifically, Gd-BA binding is accompanied by an opening of the helix-turn-helix lid together with a closure of the loops surrounding the cavity entrance (Figure 6C) [46].

The acquired relaxivity values and structural details of BABP-Gd-BA complexes constitute preliminary and promising data for the development of hepatospecific MRI CAs.

## 3. BABP as host of xanthene dyes for the development of optoelectronic devices

Host-guest encapsulation complexes are of high and wide interest in many technologically relevant applications. Bio-inspired host-guest complexes represent a new avenue of research in several fields, for example in the development of novel opto-biomaterials. The ability to bind and solubilise hydrophobic ligands, common to several protein families, can be exploited to preserve the optical properties of dyes and enable the development of emitting devices. There are a few excellent examples where biomacromolecules, able to oligomerize into nanoscaled architectures, such as fibrils and dendrimers, are employed to include guest dyes [48–50]. In other reported examples the construction of the protein-dye system was based on the formation of a covalent complex[51].

**Figure 7:**
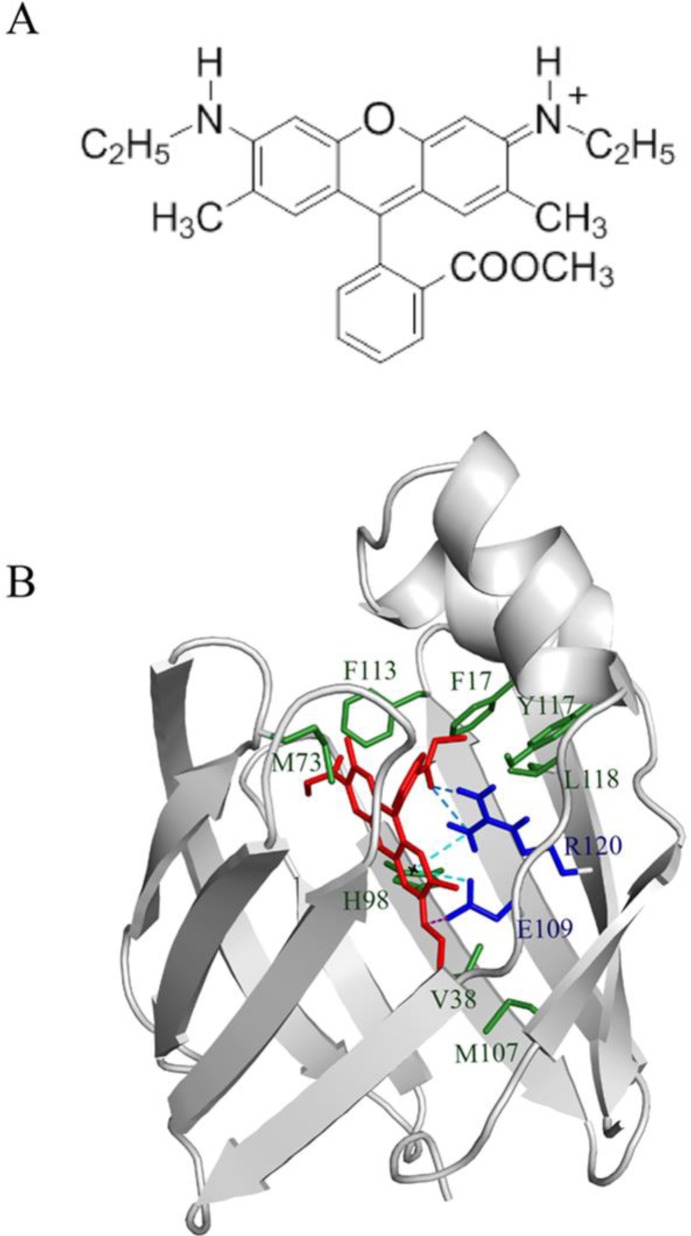
BABP-RHD host-guest complex. A) Rhodamine methyl ester chemical structure, B) BABP residues, involved in hydrophobic (green sticks) and polar (blue sticks) interactions with RHD (red) are shown. For protein residues only polar hydrogens are shown. H-bonds, π-hydrogen bonds and the salt bridge are shown as dotted blue, cyan and purple lines, respectively.

We recently described a new approach, employing BABP as a monomeric bio-matrix, to host the xanthene dye rhodamine (RHD) (Figure 7A), [52]. BABP fully encapsulates one RHD molecule inside the beta-barrel, efficiently suppressing fluorescence aggregation processes typical of the laser dye (*vide infra*). The ease of preparation of this non covalent host-guest system, by simple mixture of the components, the water-soluble character of the obtained material, processable under ambient and green conditions, are promising basis for the development of sustainable electronic and optoelectronic devices.

A further advantage of this approach is the possibility of a detailed investigation of the structural determinants of the host-guest interactions, which can be efficiently performed by NMR, in view of the optimisation of the optical properties.

The described BABP-RHD complex is characterised by a micro-molar affinity, as derived from titration experiments, based on a series of ^1^H-^15^N HSQC spectra of the protein in the presence of increasing amounts of RHD. Data driven docking studies, obtained with the HADDOCK program, highlighted the presence of many protein-dye electrostatic and hydrophobic interactions (H and -bonds, a salt bridge and hydrophobic contacts) as summarized in Figure 7B. The relevant role of the RHD ethoxy carbonyl-phenyl group in establishing specific interactions with the protein was indirectly demonstrated by studying the interactions of oxazine-4, a RHD analogue missing this functional group. Oxazine-4 indeed exhibited only very weak affinity towards the protein, as shown by the negligible chemical shift perturbation effects observed (see Supporting Information Figure S1). Thus, specific interactions involving ethoxy carbonyl-phenyl group contribute to protein-dye stability, driving the orientation of the ligand within the protein cavity.

RHD encapsulation in the protein internal cavity prevented dye-dye aggregation phenomena, usually occurring in solution at dye concentrations higher than 10^-5^ M, which are responsible for fluorescent quenching processes (photoluminescence quantum yield, QY <0.1). The photoluminescence decay of the BABP:RHD complex was mono-exponential with a lifetime of about 4 ns, typical of isolated RHD molecules in diluted solution. In view of optoelectronic applications the most important feature is whether BABP is able to preserve the good rhodamine optical properties, even in the solid state. Protein-dye films, obtained by solution casting, did not show any signature of aggregation, indicating that, even in the solid state RHD was encapsulated in the protein barrel. The QY was however drastically reduced (0.19) and the PL lifetime shortened (1.3 s). In view of the device development some efforts were made to stabilize the protein structure in the solid state through the addition of cosolutes. Good results were obtained in the presence of threalose (THR) and LiCl salts: QY indeed rose up to 0.5 and triplicated lifetimes were obtained. Film morphology, investigated by means of non-contact atomic force microscopy and fluorescence microscopy, showed that the films obtained in these conditions presented a homogeneous and flat surface, with variable thickness in the range 250-500 nm [52]. Cast films showed unchanged photophysical properties after storage time up to more than one year. Temperature stability in the range 20-50°C was ascertained by NMR. In summary, the photophysical and morphological properties of the thin films, easily obtained by water deposition, together with their stability provided a proof of concept of the viability of this bio host-guest system for the preparation of single or multilayer devices for applications in the field of optoelectronics. Bio-inspired devices, as luminescent solar concentrators, optical sensors, memories, OLEDs and solar cells will be able to benefit from the development of this class of nano-materials.

## Summary and Outlook

The discussed data support the view that BABPs are good candidates for the development of versatile bio-host systems. The capability of this protein scaffold to adapt itself to diverse amphiphilic compounds seems related to: i) the presence of key polar and hydrophobic residues, flanking the protein cavity, able to anchor different ligands; ii) a high degree of flexibility at the protein open-end. The portal area is mostly affected by complex formation, as highlighted by NMR data and essential dynamics analysis of the molecular dynamics trajectories of proteins in unbound and ligand-bound states [53]. Major concerted motions involve three contiguous structural elements of the portal area, namely the helix-loop-helix motif, the CD and EF loops, however displaying different dynamical coupling in the presence or in the absence of the ligands.

The high stability of the BABP barrel fold and the ease of recombinant protein expression, will allow the redesign of its cavity through mutagenesis, opening the way to further optimisation of protein-ligand recognition and affording host-guest systems suitable for applications in the fields of both nanomedicine and nanomaterials.

## Supplementary Material

Bile acid binding protein: a versatile host of small hydrophobic ligands for applications in the fields of MRI contrast agents and bio-nanomaterials
