# Supplementary material for: Bile acid binding protein: a versatile host of small hydrophobic ligands for applications in the fields of MRI contrast agents and bio-nanomaterials
Source: Comput Struct Biotechnol J. 2013 Dec 8;6:e201303021. doi: 10.5936/csbj.201303021 (PMC3962148; doi:10.5936/csbj.201303021)
Supplement: Bile acid binding protein: a versatile host of small hydrophobic ligands for applications in the fields of MRI contrast agents and bio-nanomaterials [file CSBJ-6-e201303021_SM0001.pdf]

Supporting Information

Bile acid binding protein: a versatile host of small hydrophobic ligands for applications in the fields of MRI contrast agents and bio-nanomaterials

Katiuscia Pagano, Simona Tomaselli, Serena Zanzoni, Michael Assfalg, Henriette Molinari, Laura Ragona

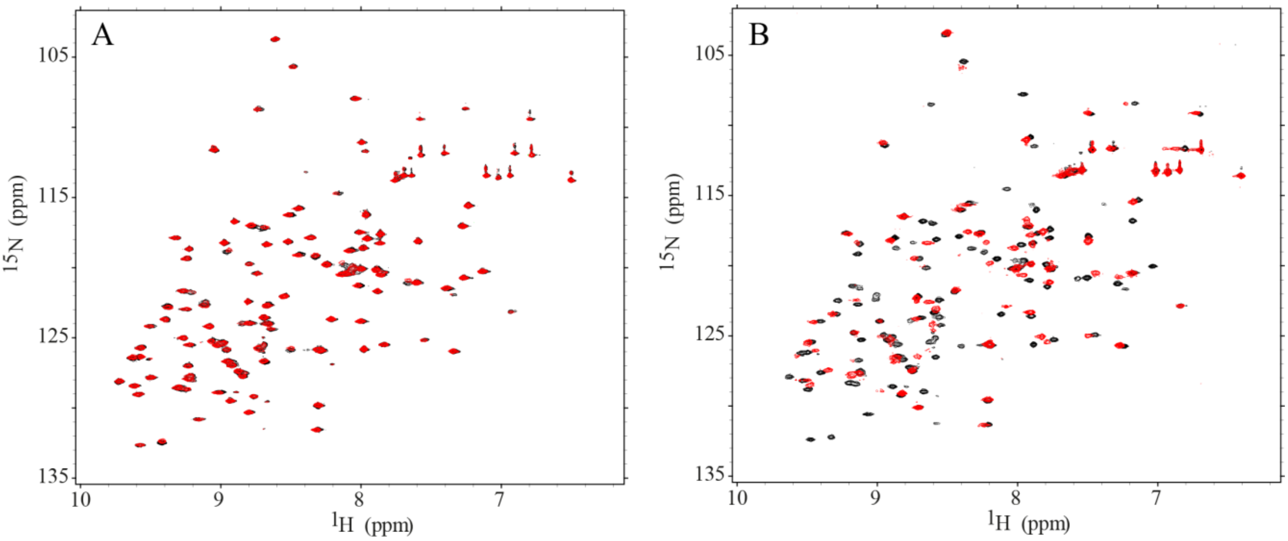

Figure S1. Superposition of ^{1}H-^{15}N HSQC spectra of apo (black) and holo BABP (red), in complex with xanthene dyes (red). Sample of BABP:Oxazine-4 1:2 molar ratio, at 500 μM protein concentration (A). Sample of BABP:RHD 1:2 molar ratio, at 125 μM protein concentration (B). All the spectra were acquired at 500.13 MHz and 298 K. It should be noted that complexation of BABP with RHD not only induces resonances shifts but also significant line broadening for residues close to the binding site.
